# Supplementary material for: Forestalled phase separation as the precursor to stripe order
Source: Nat Commun. 2025 Nov 28;16:10807. doi: 10.1038/s41467-025-66563-5 (PMC12669735; doi:10.1038/s41467-025-66563-5)
Supplement: Supplementary file 1 — Supplementary Information [file 41467_2025_66563_MOESM1_ESM.pdf]

Supplementary Information for "Forestalled Phase Separation as the Precursor to Stripe Order"

Aritra Sinha[1] and Alexander Wietek[1]

[1] *Max Planck Institute for the Physics of Complex Systems, Nöthnitzer Strasse 38, Dresden 01187, Germany*

(Dated: November 10, 2025)

In this Supplementary Information we compile additional results that support or extend the main text. Section I benchmarks our finite-temperature iPEPS by demonstrating bond-dimension convergence (Fig. S1) and tracking the temperature evolution of the peak in the charge susceptibility (Fig. S2). In Section II we analyze wider cylinders with METTS, showing convergence and representative snapshots (Fig. S3). Section III presents a full temperature sweep of density-weighted cluster-size histograms on a 32×4 cylinder (Fig. S4). Section IV probes robustness of the cluster statistics versus the threshold parameter and filling (Fig. S5), and Fig. S6 summarizes the temperature dependence via the mean cluster size. Section V disentangles ensemble versus interaction effects in the charge structure factor using grand-canonical analytics, canonical free-fermion snapshots, and interacting ($U = 10$) METTS (Fig. S7), together with the U-dependence at fixed T (Fig. S8). Section VI examines next-nearest-neighbor hopping t'; representative METTS snapshots and cluster statistics are shown in Figs. S9 and S10. Finally, Section VII addresses finite-size effects by quantifying wrap-around probabilities versus threshold c (Fig. S11), comparing it across fillings on $W = 4$ (Fig. S12), and assessing the cylinder-length dependence of cluster statistics (Fig. S13).

I. IPEPS CONVERGENCE AND TEMPERATURE DEPENDENCE OF CHARGE SUSCEPTIBILITY

In the main text Fig. 1, we perform simulations using the purification [1] technique with the iPEPS ansatz to calculate the charge susceptibility $\chi_{\text{charge}} = \frac{\partial n}{\partial \mu}$ and electron density n as functions of chemical potential μ at various temperatures. We showed that a maximum in χ_{charge} emerges starting from intermediate temperatures, indicating enhanced susceptibility towards potential phase separation. These calculations were performed with bond dimension $D = 20$. Fig. S1 shows both the χ_{charge} and n for bond dimensions $D = 20$ and $D = 30$ at temperatures $T = 0.25$ and $T = 0.125$. The close correspondence between the results obtained at $D = 20$ and $D = 30$

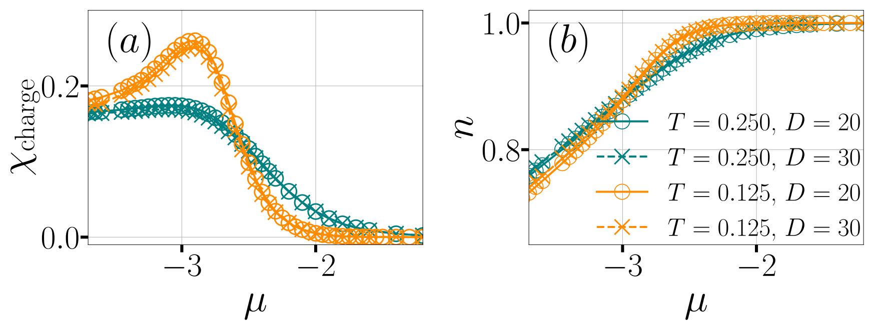

Figure S1. **Convergence of Charge Susceptibility and Density with Bond Dimension.** (a) Charge susceptibility, $\chi_{\mathrm{charge}} = \frac{dn}{d\mu}$, and (b) filling n as functions of chemical potential μ for bond dimensions $D = 20$ (circles) and $D = 30$ (crosses), at temperatures $T = 0.25$ (teal) and $T = 0.125$ (dark orange). This demonstrates that features highlighted in main text Fig. 1 such as the peak in susceptibility have converged.

indicates that our calculations are reliably converged with respect to bond dimension, affirming the robustness of the features observed in χ_{charge}.

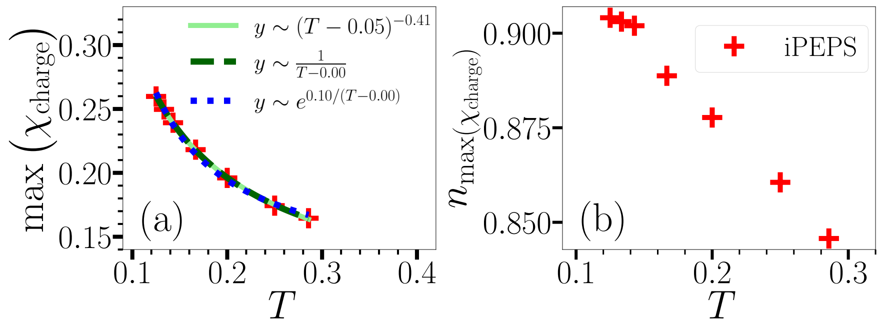

Figure S2. **Analysis of Peak Charge Susceptibility.** (a) The peak value in charge susceptibility χ_{charge} as a function of temperature. Power-law fit with $(T - T')^{\gamma}$ (solid green line) and linear fit with $1/(T - T')$ (dashed dark green line) capture a rapid growth and do not exclude a divergence of χ_{charge} as $T \to T_c \geq 0$. (b) Filling corresponding to the maximum charge susceptibility, $n_{\max(\chi_{\mathrm{charge}})}$ as a function of temperature T.

Fig. S2(a) displays the peak charge susceptibility $\max\left(\chi_{\mathrm{charge}}\right)$ as a function of temperature. Upon cooling, $\max\left(\chi_{\mathrm{charge}}\right)$ rises steadily; the trend can be fitted equally well by a Curie-Weiss-like form $1/(T - T')$ and by a power-law $(T - T')^{\gamma}$. The good agreement of both fits with the numerical points implies that χ_{charge} may diverge as T approaches a critical value $T_c \geq 0$, signaling a strong enhancement of charge fluctuations that we attribute to incipient hole clustering. Such clustering serves as a precursor to phase separation; however, as discussed in the main text, the onset of stripe order at lower temperatures ultimately prevents full phase separation. In Fig. S2(b), we plot the density corresponding to the maximum of charge susceptibility, $n_{\max(\chi_{\mathrm{charge}})}$ as a function of

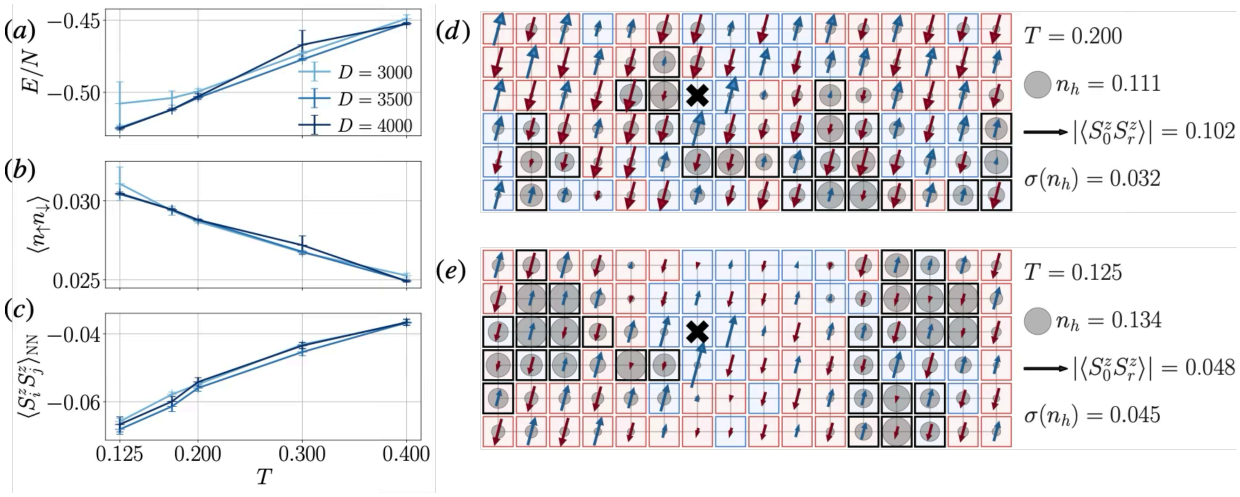

Figure S3. **Convergence of larger width cylinder and METTS snapshots**. (a) Energy per site E/N, (b) double occupancy $\langle n_\uparrow n_\downarrow \rangle$ and (c) nearest-neighbor spin-spin correlation $\langle S^z_i S^z_j \rangle_{\rm NN}$ versus temperature T on a cylinder of length $L = 16$ and width $W = 6$ at filling $n = 0.9375$ and on-site repulsion $U = 10$ at various bond dimensions D. The data are well converged up to $T = 0.125$. The error bars reflect 1σ uncertainty. Typical METTS snapshots for same parameters at temperatures (d) $T = 0.2$ and (e) $T = 0.125$ showing hole clustering and antiferromagnetic domains, similar to observations on narrower cylinders.

temperature.

II. CONVERGENCE FOR WIDER METTS CYLINDERS

In the main text, we used METTS simulations to study the formation of hole clusters at intermediate temperatures ($0.5 \lesssim T \lesssim 0.075$) and the emergence of stripe order at very low temperatures ($T \lesssim 0.05$) on cylinders of width $W = 4$. To check whether these features persist at larger widths, we perform additional METTS simulations on a 16×6 cylinder. Converging a width-6 system with matrix product states (MPS) is more challenging because the entanglement entropy S across a bipartition scales as $S \propto W$ in two-dimensional systems represented by one-dimensional MPS [2]. In practice, $S \approx \alpha W$, so the required bond dimension grows like $D \propto e^S \approx e^{\alpha W}$ [3].

Fig. S3 shows (a) the energy per site, (b) the average double occupancy and (c) nearest-neighbor spin-spin correlation $\langle S^z_i S^z_j \rangle_{\rm NN}$ versus temperature for the 16×6 cylinder at electron density $n = 0.9375$ and on-site repulsion $U = 10$ using bond dimensions $D = 3000, 3500, 4000$. The close overlap down to $T = 0.125$ indicates convergence. In Fig. S3(d) and (e), we have representative METTS snapshots for $T = 0.2$ and $T = 0.125$. They show strong hole clustering and large antiferromagnetic domains. Black-bordered sites mark hole densities above the threshold in Eq. 4

of the main text (or Eq. S1 here with $c = 0.5$), and adjacent black-bordered sites are grouped into nearest–neighbor clusters $\mathcal{C}$ [Eq. 7 in the main text]; for each cluster we record its size $m = |\mathcal{C}|$ and hole mass $\rho(\mathcal{C}) = \sum_{r\in\mathcal{C}} n_h(r)$. We recall the density–weighted size distribution (Eq. 10 and Eq. 11 in the main text)

$$p_m = \frac{\sum_{\text{snapshots}} \sum_{\mathcal{C}:\,|\mathcal{C}|=m} \rho(\mathcal{C})}{\sum_{\text{snapshots}} \sum_{\mathcal{C}} \rho(\mathcal{C})}, \qquad \sum_m p_m = 1,$$

its hole–mass decomposition $p_m = \sum_{I\in\mathcal{I}} p_m^{(I)}$.

III. CLUSTERING STATISTICS FOR A WIDE RANGE OF TEMPERATURES

As a comprehensive analysis of how hole clustering evolves across the entire temperature range studied, Fig. S4 systematically presents the temperature-dependent evolution of the stacked histograms of density-weighted cluster-size distributions, p_m vs cluster size m (described in detail in the main text), obtained from METTS simulations on a 32×4 cylinder at strong coupling ($U = 10$) and a fixed doping of $n = 0.9375$. At the highest temperatures ($T \geq 1.0$), hole clusters are small and their sizes are exponentially suppressed, reflecting largely uncorrelated holes. Upon reducing the temperature into the intermediate regime ($T = 0.5$ down to $T = 0.075$), the distributions distinctly broaden, signaling the emergence of extended hole clusters. Finally, at the lowest temperatures ($T \leq 0.05$), the distribution becomes sharply peaked around a characteristic cluster size of $m = 12$, marking the onset of a well-defined charge density wave (stripe-ordered) phase.

IV. SENSITIVITY OF THE CLUSTER DEFINITION AND ROBUSTNESS ACROSS FILLINGS

In the main text, hole clusters in METTS snapshots are identified by thresholding the local hole density,

$$n_h^{\text{th}} \;=\; 1 - n + c\,\sigma_{n_h}, \tag{S1}$$

where n is the average electron density, σ_{n_h} is the snapshot standard deviation of the hole density (Eq. 5 of the main text), and c is a tunable coefficient (set to 0.5 in the main text). We analyze the resulting cluster-size statistics and their temperature dependence to see the robustness of the clustering signal.

Figure S5 top row shows the density–weighted cluster–size distribution p_m at temperature $T = 0.10$ on a 32×4 cylinder for $c \in \{0,\, 0.35,\, 0.70,\, 1.00\}$ at $n = 0.9375$. Increasing c makes the threshold

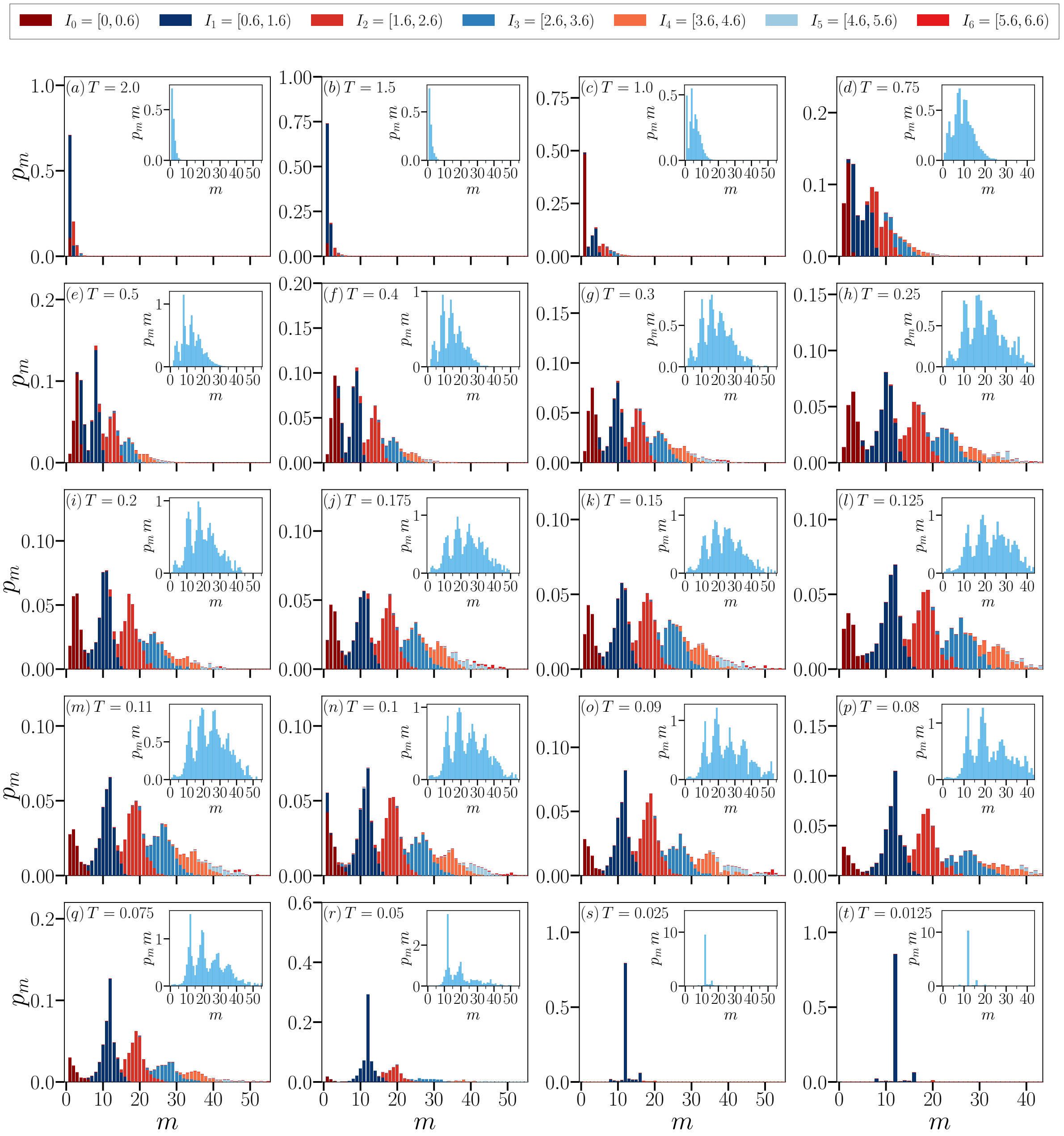

Figure S4. **Evolution of cluster-size distributions in METTS snapshots across temperatures.** Stacked histograms of the density–weighted cluster–size distribution p_m vs. size m for a 32×4 cylinder at $U = 10$, $n = 0.9375$. Colors encode the cluster hole mass $\rho(\mathcal{C})$ binned into $\mathcal{I} = \{[0, 0.6), [0.6, 1.6), [1.6, 2.6), [2.6, 3.6), [3.6, 4.6), [4.6, 5.6)\}$, so $p_m = \sum_{I \in \mathcal{I}} p_m^{(I)}$. Insets in each panel show $p_m m$. Panels (ordered left-to-right, top-to-bottom): (a) $T = 2.0$, (b) $T = 1.5$, (c) $T = 1.0$, (d) $T = 0.75$, (e) $T = 0.5$, (f) $T = 0.4$, (g) $T = 0.3$, (h) $T = 0.25$, (i) $T = 0.2$, (j) $T = 0.175$, (k) $T = 0.15$, (l) $T = 0.125$, (m) $T = 0.11$, (n) $T = 0.1$, (o) $T = 0.09$, (p) $T = 0.08$, (q) $T = 0.075$, (r) $T = 0.05$ (s) $T = 0.025$, (t) $T = 0.0125$. At high T panels (a–d) large clusters are exponentially suppressed; at intermediate T panels (e–q) the cluster distribution broaden markedly; and at the lowest T panels (r–t) develop a pronounced peak near $m = 12$, marking stripe order.

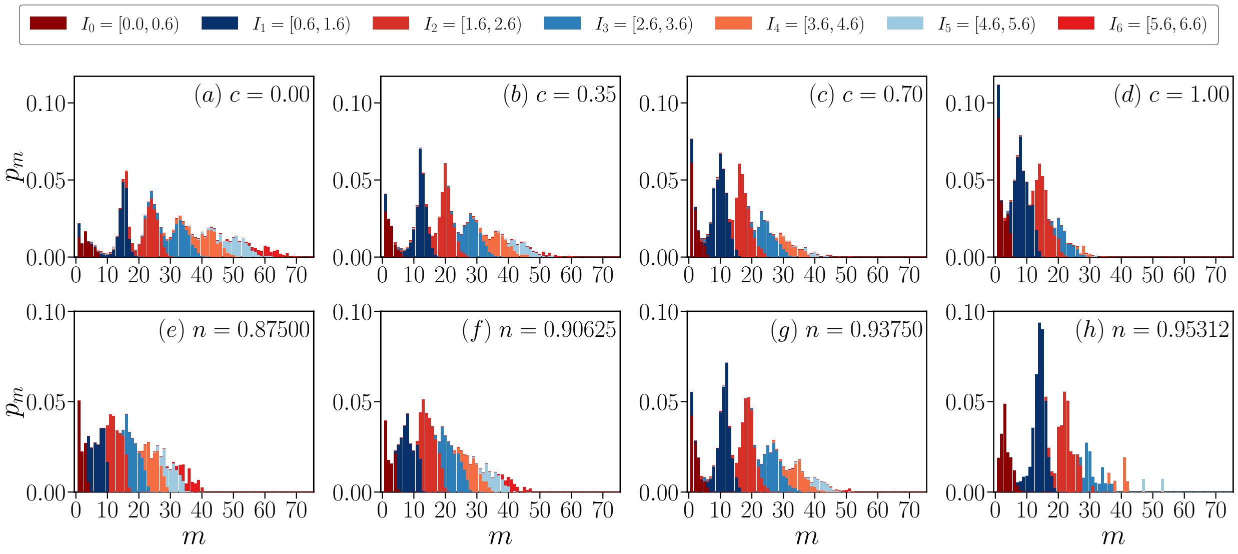

Figure S5. **Robustness of cluster statistics at fixed $T = 0.10$ on a 32×4 cylinder. Top row (threshold scan at fixed $n = 0.9375$):** (a) $c = 0$, (b) $c = 0.35$, (c) $c = 0.70$, (d) $c = 1.00$. Larger c tightens the threshold, shifting p_m lobes to lower m and reducing large clusters, yet a broad distribution and near-integer aggregation persist. **Bottom row (filling scan at fixed $c = 0.5$):** (e) $n = 0.875$, (f) $n = 0.90625$, (g) $n = 0.9375$, (h) $n = 0.95312$. Stronger AFM background above $n \approx 0.93$ (panels g–h) correlates with a clear lobe pattern and near-integer aggregation, while reduced AFM at lower n (panels e–f) yields smoother p_m with more diffuse puddles. All panels show stacked histograms of the density-weighted cluster-size distribution p_m versus size m; colors encode the hole-mass windows $\rho(\mathcal{C})$ used throughout.

more selective, and as a consequence larger clusters form less readily and the lobe peaks in p_m shift to lower m. Crucially, however, the qualitative structure is stable: the large distribution of cluster sizes persists. The lobe-like pattern is also present and the dominant mass windows I_k still progress in near-integer steps as m increases. Thus, while c controls how tightly we crop clusters, the broad spread in cluster sizes at intermediate temperature and near-integer aggregation pattern for low doping is not an artifact of choosing $c = 0.5$.

In Fig. S5 bottom row we fix $c = 0.5$ and temperature $T = 0.10$ and scan the filling $n \in \{0.875, 0.90625, 0.9375, 0.95312\}$ on the same 32×4 cylinder. When we decrease the filling below $n \simeq 0.93$, it weakens the antiferromagnetic background that stabilizes sharp domain walls, which suppresses the oscillatory, lobe-like structure. In short, stronger AFM (above $n \approx 0.93$) correlates with coherent near-integer aggregation, whereas weaker AFM (e.g., $n = 0.875$) yields more diffuse puddles and a smoother p_m.

To complement the fixed-T comparison, we also track the mean cluster size $\bar{m}(T)$ (Eq. 12 of the main text). Figure S6(a) shows $\bar{m}(T)$ at $n = 0.9375$ for several c; all choices display the same

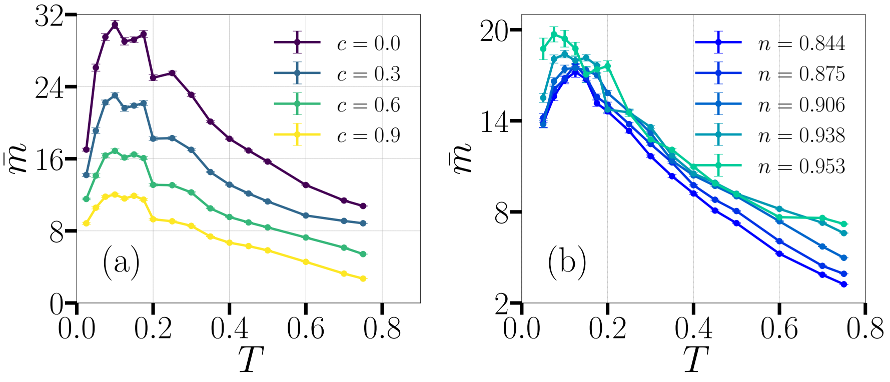

Figure S6. **Robustness of the mean cluster size.** Cylinder $L = 32$, $W = 4$, $U = 10$. (a) $\bar{m}(T)$ at $n = 0.9375$ for several c in Eq. (S1); all curves show the same overall thermal trend. (b) $\bar{m}(T)$ at fixed $c = 0.5$ across $n \in [0.84375, 0.95312]$, demonstrating robust clustering at intermediate temperatures. The error bars reflect 1σ uncertainty.

qualitative evolution, with small clusters at high T, a broad enhancement at intermediate T, and a selected size at the lowest T. Figure S6(b) demonstrates that this intermediate-T clustering persists across a wide density range, $n \in [0.84375, 0.95312]$ (bond dimension $D = 2000$ throughout). This window spans both strange-metal and pseudogap regimes, indicating that finite-T charge clustering is a robust and broadly reproducible feature of the model.

V. CHARGE STRUCTURE FACTOR: ENSEMBLE AND INTERACTION DEPENDENCIES

In the main text (Fig. 5) we defined the charge structure factor

$$S_c(\mathbf{k}) = \frac{1}{N}\sum_{l,m} e^{i\mathbf{k}\cdot(\mathbf{r}_l - \mathbf{r}_m)}\langle (n_l - n)(n_m - n)\rangle, \tag{S2}$$

which exhibits an inner peak at $\mathbf{k} = (2\pi/L, 0)$ for METTS simulations done in the canonical ensemble at strong coupling. In the canonical ensemble the total density is fixed, so by construction $S_c(\mathbf{k} = 0) = 0$. If the inner peak at the smallest nonzero momentum were purely an artifact of enforcing $S_c(0) = 0$, one would observe a small but broad redistribution of weight toward $k_x = 2\pi/L$ even in the non-interacting limit.

To understand this, we compare the $U = 10$ METTS results with canonical and grand-canonical results for the free-fermion case ($U = 0$) for a 32×4 cylinder. To transform Eq. S2 into momentum

space, we introduce

$$c_{\mathbf{k}\sigma} = \frac{1}{\sqrt{N}} \sum_j e^{-i\,\mathbf{k}\cdot\mathbf{r}_j}\, c_{j\sigma} \qquad \text{(with } N = LW \text{ sites)} \tag{S3}$$

so that

$$\hat{n}_l = \sum_\sigma c^\dagger_{l\sigma}\, c_{l\sigma} = \frac{1}{N} \sum_{\mathbf{k},\,\mathbf{p},\,\sigma} e^{\,i\,(\mathbf{k}-\mathbf{p})\cdot\mathbf{r}_l} c^\dagger_{\mathbf{p}\sigma}\, c_{\mathbf{k}\sigma}. \tag{S4}$$

Define the Fourier transform of the density fluctuation,

$$\delta\hat{n}_{\mathbf{q}} \equiv \sum_l e^{\,i\,\mathbf{q}\cdot\mathbf{r}_l}\, (\hat{n}_l - n) = \sum_{\mathbf{k},\,\sigma} c^\dagger_{\mathbf{k}+\mathbf{q},\sigma}\, c_{\mathbf{k}\sigma} - nN\delta_{\mathbf{q},\mathbf{0}}, \tag{S5}$$

where the geometric sum $\sum_l e^{\,i\,(\mathbf{q}+\mathbf{k}-\mathbf{p})\cdot\mathbf{r}_l} = N\,\delta_{\mathbf{q}+\mathbf{k}-\mathbf{p},\,\mathbf{0}}$ forces $\mathbf{p} = \mathbf{k}+\mathbf{q}$, and $\delta_{\mathbf{q},\mathbf{0}}$ is the Kronecker delta in momentum space. Equation (S2) can now be written compactly as

$$S_c(\mathbf{q}) = \frac{1}{N} \left\langle \delta\hat{n}^\dagger_{\mathbf{q}}\, \delta\hat{n}_{\mathbf{q}} \right\rangle \tag{S6}$$

For a non-interacting system, the many-body state is Gaussian, so Wick's theorem reduces any four-fermion average to products of two-fermion ones. With $f_{\mathbf{k}} = [e^{\beta(\varepsilon_{\mathbf{k}}-\mu)} + 1]^{-1}$ and $\varepsilon_{\mathbf{k}} = -2t(\cos k_x + \cos k_y)$,

$$\left\langle \hat{c}^\dagger_{\mathbf{k}\sigma} \hat{c}_{\mathbf{k}+\mathbf{q},\sigma}\, \hat{c}^\dagger_{\mathbf{p}\sigma'} \hat{c}_{\mathbf{p}-\mathbf{q},\sigma'} \right\rangle_{\rm GC} = \delta_{\mathbf{k},\mathbf{p}-\mathbf{q}}\, \delta_{\sigma\sigma'}\; f_{\mathbf{k}}(1 - f_{\mathbf{k}+\mathbf{q}}).$$

Inserting this into (S6), summing over the two spin projections, and noting that the $-nN\delta_{\mathbf{q},\mathbf{0}}$ term in (S5) cancels *only* for $\mathbf{q} = \mathbf{0}$, one obtains

$$S_c^{\rm GC}(\mathbf{q}) = \frac{2}{N} \sum_{\mathbf{k}} f_{\mathbf{k}} \big[1 - f_{\mathbf{k}+\mathbf{q}}\big]. \tag{S7}$$

The chemical potential μ is fixed at each T by the single bisection condition $\frac{2}{LW} \sum_{\mathbf{k}} f_{\mathbf{k}} = n$. Next for the charge structure in the fixed-N sector (canonical), we start from the grand-canonical one-body correlator

$$C_{ij} = \langle \hat{c}^\dagger_j \hat{c}_i \rangle_{\rm GC} = \sum_{\mathbf{k}} \varphi_{i\mathbf{k}}\, f_{\mathbf{k}}\, \varphi^*_{j\mathbf{k}}, \qquad \varphi_{i\mathbf{k}} = N^{-1/2} e^{i\mathbf{k}\cdot\mathbf{r}_i}. \tag{S8}$$

Because a free system is Gaussian, C alone fixes the density matrix $\rho_{\rm GC} = \mathcal{N} \exp[-\sum_{ij}(\ln[(1 - C)C^{-1}])_{ij} \hat{c}^\dagger_i \hat{c}_j]$ [4]. We transform the grand-canonical state into statistically exact N-particle Slater determinants by a single measurement sweep:

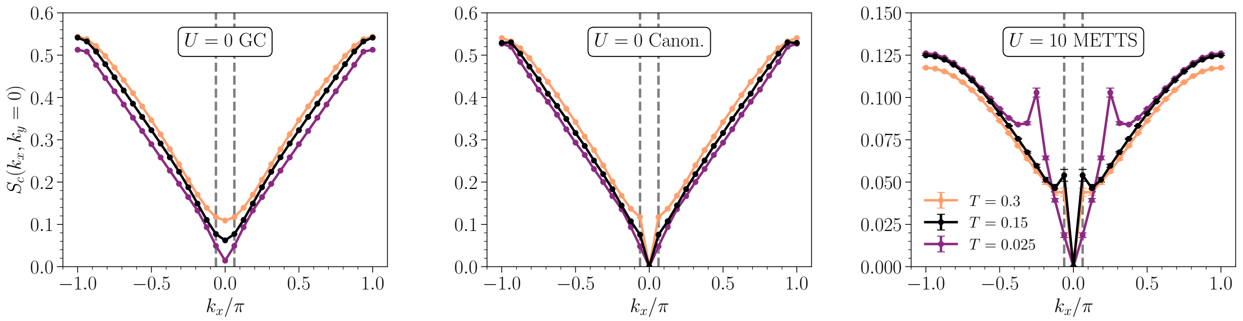

Figure S7. **Charge Structure Factor — METTS vs free-fermion comparisons.** Charge–structure factor at $n = 0.9375$ for a cylindrical lattice of size $L = 32, W = 4$. **Left:** analytic grand–canonical result **Center:** canonical result from free-fermion snapshots. **Right:** interacting $U = 10$ METTS. Only the interacting case exhibits the subtle inner peak at $k_x = 2\pi/L$ for intermediate temperatures $T = 0.3, 0.15$ and a sharp peak at $k_x = \pi/4$ for the charge-density-wave at $T = 0.025$. The presence of the inner peak at intermediate temperatures uniquely in the interacting ($U = 10$) canonical METTS data suggests genuine emergent long-wavelength density correlations rather than numerical artifacts or trivial ensemble effects. The error bars reflect 1σ uncertainty.

1. For site i draw $n_i \in \{0, 1\}$ with probability $p_i(1) = C_{ii}$ and $p_i(0) = 1 - C_{ii}$.

2. Conditionally update the correlator

$$C \longrightarrow \begin{cases} C - \dfrac{|v\rangle\langle v|}{p_i(1)}, & n_i = 1,\ v_j = C_{ji} \\ C + \dfrac{|u\rangle\langle u|}{p_i(0)}, & n_i = 0,\ u_j = \delta_{ji} - C_{ji} \end{cases} \tag{S9}$$

3. Record the occupation $O_i \equiv n_i$ and continue until all sites are visited.

4. Accept the snapshot $\{O_i\}$ only if $\sum_i O_i = N = nLW$; otherwise reject.

For every *accepted* snapshot α define the Fourier transform of the density deviation

$$\widetilde{n}^{(\alpha)}(\mathbf{q}) = \sum_j e^{i\mathbf{q}\cdot\mathbf{r}_j} \left(O_j^{(\alpha)} - n\right), \tag{S10}$$

which satisfies $\widetilde{n}^{(\alpha)}(\mathbf{0}) = 0$ exactly because the particle number is fixed. Since the $O_j^{(\alpha)}$ are ordinary numbers, operator ordering issues are absent and

$$|\widetilde{n}^{(\alpha)}(\mathbf{q})|^2 = \sum_{l,m} e^{i\mathbf{q}\cdot(\mathbf{r}_l - \mathbf{r}_m)} (O_l^{(\alpha)} - n)(O_m^{(\alpha)} - n) \tag{S11}$$

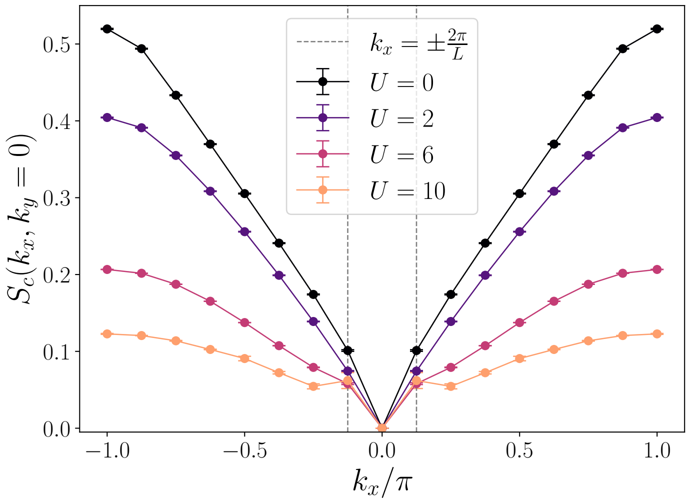

Figure S8. **Charge Structure Factor for different Interaction Strengths.** Charge structure factor $S_c(k_x, k_y = 0)$ for a cylindrical lattice of size $L = 16, W = 4$ cylinder at $T = 0.15$ obtained with METTS in the canonical ensemble. The non-interacting system ($U = 0$) shows no inner peak; only at strong coupling ($U = 10$) does a clear, though still modest, tiny peak emerge at the smallest non-zero momentum $k_x = \pm 2\pi/L$. The emergence of a small peak exclusively at strong coupling ($U = 10$) unambiguously connects observed density correlations to interaction-driven physics, rather than trivial finite-size or ensemble effects. The error bars reflect 1σ uncertainty.

is the desired integrand of the two-point correlator. Averaging over all accepted snapshots yields the canonical structure factor

$$S_c^{\mathrm{C}}(\mathbf{q}) = \frac{1}{N} \left\langle \left| \widetilde{n}^{(\alpha)}(\mathbf{q}) \right|^2 \right\rangle_\alpha . \tag{S12}$$

Fig. S7 gathers the three data sets side by side. The grand-canonical curve (left panel, analytic result (S7)) is featureless once the trivial $q = 0$ weight is excluded, while the canonical snapshot average (center panel, estimator above) differs from it only by the expected $\mathcal{O}(1/N)$ finite-size corrections. Indeed, at $T = 0.15$ the two free-fermion spectra are already indistinguishable beyond the first momentum point, and at the lowest temperature $T = 0.025$ become almost indistinguishable within plotting resolution.

In stark contrast, the interacting $U = 10$ METTS data (right panel) display a narrow “inner” peak at $k_x = 2\pi/L$ for $T = 0.30$ and 0.15, a feature that *cannot* be reproduced by either free-fermion ensemble. Upon further cooling to $T = 0.025$ this weight is transferred almost entirely to a dominant peak at $k_x = \pi/4$, signaling the formation of a period-8 charge–density wave. Their absence in the free-fermion curves confirms that both peaks are interaction driven.

Finally, we perform METTS simulations on an $L \times W = 16 \times 4$ cylinder at $T = 0.15$ for $U = 0, 2, 6$, and 10. Convergence was reached with bond dimension $D_{\max} = 3000$; for $U = 0$ the resulting S_c almost coincides with the direct free-fermion snapshots discussed earlier. As shown in Fig. S8, the $U \leq 4$ curves remain essentially undisturbed at $k_x = 2\pi/L$, whereas only at $U = 10$ does a small peak develop and a barely discernible shoulder is present at the intermediate $U = 6$ as well. This demonstrates that canonical-ensemble weight redistribution alone cannot generate the observed structure–factor peak; its emergence at strong coupling reflects genuine long–wavelength density correlations and a tendency towards phase separation.

VI. EFFECT OF NEXT–NEAREST–NEIGHBOR HOPPING (t') ON HOLE CLUSTERING

The square-lattice Hubbard model studied here includes diagonal hopping t' in addition to the nearest-neighbor amplitude t:

$$\hat{H} = -t \sum_{\langle i,j \rangle,\sigma} \left(c^{\dagger}_{i\sigma} c_{j\sigma} + \text{H.c.} \right) - t' \sum_{\langle\langle i,j \rangle\rangle,\sigma} \left(c^{\dagger}_{i\sigma} c_{j\sigma} + \text{H.c.} \right) + U \sum_{i} n_{i\uparrow} n_{i\downarrow}. \tag{S13}$$

The results presented in the main text were obtained with zero next–nearest–neighbor hopping ($t' = 0$) and a lightly doped filling $n = 0.9375$. In order to disentangle the roles of kinetic frustration and mobile holes, we perform METTS simulations at a finite value $t' = 0.3$ for two electronic densities: the same doped case as before and, the half-filled Mott insulator $n = 1$. All calculations were done at interaction strength $U = 10$ on 32×4 cylinders, a TDVP time step $\Delta\tau = 0.2$, a maximum bond dimension $D_{\max} = 3000$, and discard the first few METTS iterations to suppress autocorrelation. Two temperatures are studied $T = 0.20$ and $T = 0.15$ similar to those in the main text.

Fig. S9 contrasts representative METTS snapshots at those temperatures. The lightly doped system at filling $n = 0.9375$ for temperatures (c) $T = 0.2$ and (d) $T = 0.15$ (bottom row) displays the familiar pattern of hole-rich clusters surrounded by antiferromagnetic domains. The half-filled case ($n = 1$, top row) for temperatures (a) $T = 0.2$ and (b) $T = 0.15$ remains essentially uniform: staggered spin correlations alternate regularly, with no sign of segregation.

Cluster–size histograms of $p_m m$ vs m for temperatures $T = 0.2$ and $= 0.15$ in Fig. S10 akin to inset of Fig. S4 confirm this contrast: at $n = 1$ (top row) the distribution is short-ranged, while at $n = 0.9375$ (bottom row) it acquires a broad tail. Within the present parameter set, the half-filling remains homogeneous, indicating that kinetic frustration alone is insufficient; mobile holes

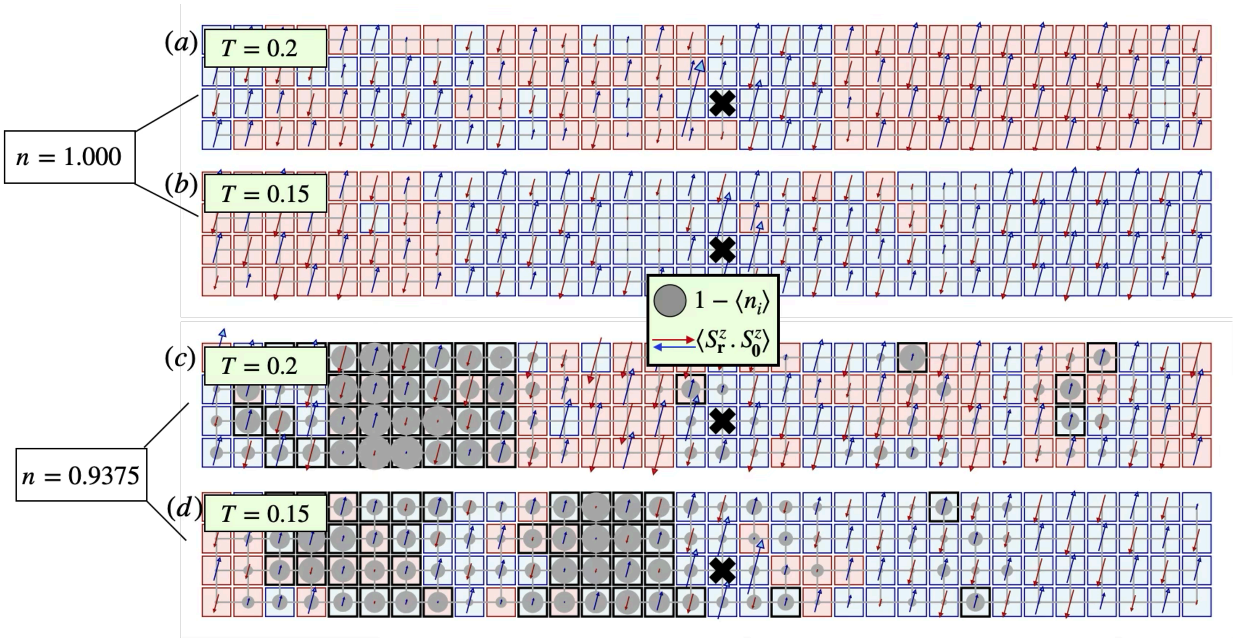

Figure S9. **METTS snapshots for $t' = 0.3$ Hubbard model.** The typical METTS snapshots are for a cylinder of length $L = 32$ and $W = 4$, at on-site repulsion $U = 10$. The top snapshot at filling $n = 1$ for temperatures (a) $T = 0.2$ and (b) $T = 0.15$ shows no clustering and bottom is at filling $n = 0.9375$ for temperatures (c) $T = 0.2$ and (d) $T = 0.15$ shows hole clustering. The circle area is proportional to the hole density, arrows show staggered S^zS^z correlations. Clustering appears only at finite doping.

are required to nucleate charge clusters. A thorough exploration of varying t' and its interplay with doping and temperature will be addressed comprehensively in future work.

VII. ADDRESSING FINITE SIZE CYLINDER EFFECTS

Hole clusters on a finite-width cylinder can, in principle, encircle the periodic transverse boundary and artificially affect the p_m distribution. We say that a cluster "wraps" around the cylinder if it contains at least one site on the first transverse edge ($y = 0$) and at least one site on the opposite edge ($y = W - 1$). To quantify this, we compute the wrap–around probability

$$P_{\text{wrap}}(T) = \frac{N_{\text{wrap}}(T)}{N_{\text{tot}}(T)},$$

where $N_{\text{tot}}(T)$ is the total number of clusters identified at temperature T, and $N_{\text{wrap}}(T)$ is the number of those clusters that wrap.

We evaluate $P_{\text{wrap}}(T)$ for threshold coefficients (see Eq. S1) $c = 0.0, 0.5, 1.0$ on 32×4 cylinders at maximum bond dimension $D = 2000$ and on 16×6 cylinders at $D = 3000$ and $D = 4000$. Fig. S11 shows that for all c, $P_{\text{wrap}}(T) < 0.70$ for $T \gtrsim 0.05$ on $W = 4$, and $P_{\text{wrap}}(T) \lesssim 0.50$ for

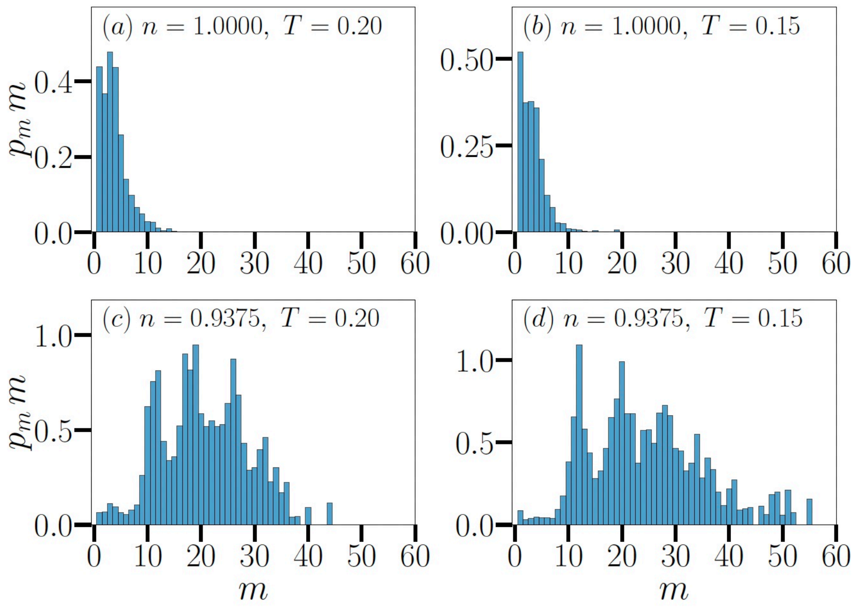

Figure S10. **Cluster-size statistics for $t'/t = 0.3$ Hubbard model.** Product of density-weighted probability and cluster size, $p_m m$ vs. cluster size m for filling $n = 1$ (temperatures (a) $T = 0.2$ and (b) $T = 0.15$) and for filling $n = 0.9375$ (temperatures (c) $T = 0.2$ and (d) $T = 0.15$). Broad tails appear only in the doped case. The simulations are conducted on a $L = 32$ and $W = 4$ cylinder at on-site repulsion $U = 10$.

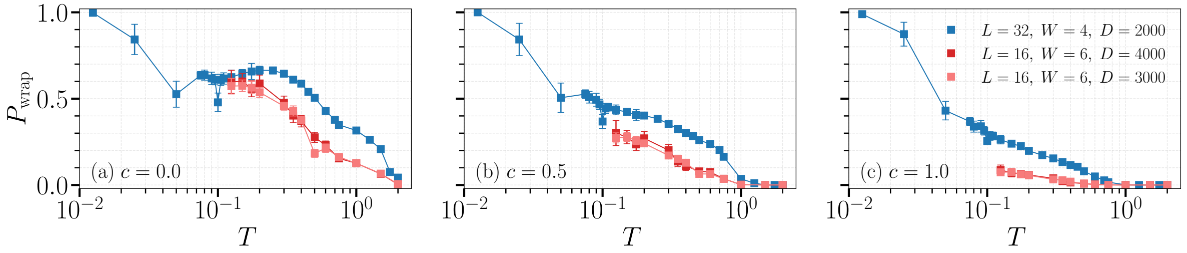

Figure S11. **Wrap-around probability $P_{\rm wrap}(T)$ of hole clusters.** Panels (a–c) show $P_{\rm wrap}(T)$ for threshold coefficients (a) $c = 0.0$, (b) $c = 0.5$, and (c) $c = 1.0$. The results are obtained on 32×4 cylinders at bond dimension $D = 2000$ (blue) and on 16×6 cylinders at $D = 3000$ (orange) and $D = 4000$ (red). Error bars indicate 1σ uncertainty. For all c, $P_{\rm wrap}(T)$ remains below 0.7 for $T \gtrsim 0.05$ on $W = 4$ and below 0.5 for $T \gtrsim 0.10$ on $W = 6$, showing that most clusters are localized at intermediate temperatures. At the lowest temperatures $P_{\rm wrap}$ approaches unity, signaling the emergence of system-spanning stripe domains.

$T \gtrsim 0.10$ on $W = 6$. For the $c = 0.5$ used in the main text, $P_{\rm wrap}(T) < 0.50$ for $T \gtrsim 0.05$ on $W = 4$, and $P_{\rm wrap}(T) \lesssim 0.30$ for $T \gtrsim 0.10$ on $W = 6$. Thus a non-negligible fraction of clusters do not wrap in the forestalled phase-separation regime. Only below $T \approx 0.02$, coinciding with stripe order, does $P_{\rm wrap}(T)$ rise steeply to unity, consistent with stripe domains spanning the cylinder.

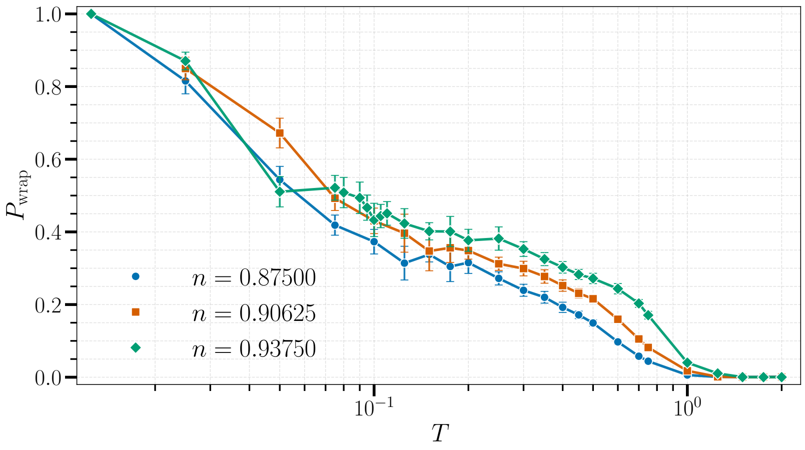

Figure S12. **Wrap–around probability with different fillings.** Probability that a cluster wraps the transverse direction, $P_{\rm wrap}(T)$, on a 32×4 cylinder at $U = 10$ for fillings $n \in \{0.875,\ 0.90625,\ 0.93750\}$ and $c = 0.5$. In the intermediate-T clustering window $P_{\rm wrap} < 1$ and is systematically lower at larger hole doping $1 - n$; upon further cooling, $P_{\rm wrap}$ increases toward unity as static stripes form. The error bars reflect 1σ uncertainty.

To complement Fig. S11, we compare the transverse wrap–around probability $P_{\rm wrap}(T)$ across fillings $n \in \{0.875,\ 0.90625,\ 0.93750\}$ on $L \times W = 32 \times 4$ using the same clustering rule (threshold $n_h^{\rm th} = 1 - n + 0.5\,\sigma_{n_h}$, with $c = 0.5$). As shown in Fig. S12, $P_{\rm wrap}$ in the clustering window is well below unity and decreases as we lower the filling from $n = 0.9375$ toward $n = 0.875$. This is consistent with formation of smaller clusters on $W = 4$; on further cooling toward the stripe regime, $P_{\rm wrap}$ rises toward unity.

These findings have two important physical consequences. First, the smaller wrap–around probability at intermediate temperatures confirms that the observed large clusters reflect genuine, localized hole-rich domains rather than an artifact of the periodic cylinder. Wrap-around probabilities decrease with width, so the observed hole clustering at intermediate temperatures cannot be attributed to meandering stripes. Such local clustering supports the picture of forestalled phase separation: hole droplets grow with cooling but remain finite until stripe correlations set in. Second, the sharp crossover to $P_{\rm wrap} \approx 1$ at low T signals the formation of system-spanning stripes, in agreement with the emergence of long-range spatial order.

To disentangle finite length from finite width effects, we scan $L = 16, 24, 32, 40$ at fixed $W = 4$ for $n = 0.9375$ (doping $1-n = 1/16$) and $U = 10$, using exactly the clustering protocol described in the main text. We see in Fig. S13, for $T = 0.300, 0.150, 0.100$ (all within the clustering/forestalled PS window), the distributions are qualitatively similar: p_m has a multi–lobe structure, and lowering T

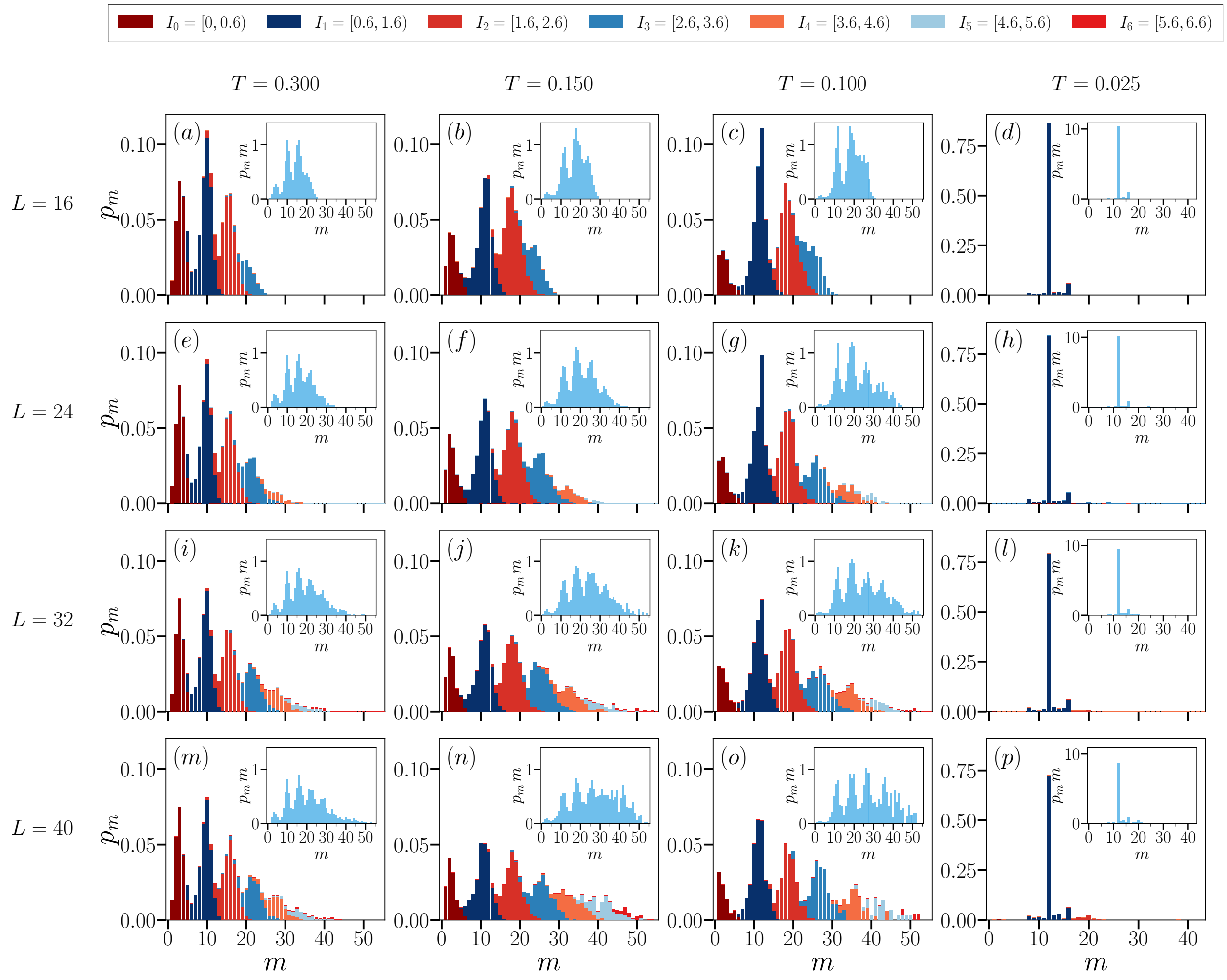

Figure S13. **Cluster statistics resolved by hole mass.** Panels (a–p) show stacked, hole-mass–resolved cluster-size distributions p_m versus m for width $W = 4$, interaction $U = 10$, and filling $n = 0.9375$. Each panel corresponds to a specific combination of system length L and temperature T: (a) $L = 16$, $T = 0.300$; (b) $L = 16$, $T = 0.150$; (c) $L = 16$, $T = 0.100$; (d) $L = 16$, $T = 0.025$; (e) $L = 24$, $T = 0.300$; (f) $L = 24$, $T = 0.150$; (g) $L = 24$, $T = 0.100$; (h) $L = 24$, $T = 0.025$; (i) $L = 32$, $T = 0.300$; (j) $L = 32$, $T = 0.150$; (k) $L = 32$, $T = 0.100$; (l) $L = 32$, $T = 0.025$; (m) $L = 40$, $T = 0.300$; (n) $L = 40$, $T = 0.150$; (o) $L = 40$, $T = 0.100$; (p) $L = 40$, $T = 0.025$. At $T = 0.300, 0.150$, and 0.100 (panels a–c, e–g, i–k, m–o), the distributions exhibit a multi-lobe structure characteristic of the clustering or forestalled-phase-separation regime, with the weight shifting gradually toward larger m as T decreases. At $T = 0.025$ (panels d, h, l, p), all lengths collapse near $m \simeq 12$, consistent with period-8 stripe order.

shifts weight slightly toward larger m without changing the overall pattern. Increasing L at fixed $W = 4$ increases the total hole number $N_h = (1 - n)LW$ ($N_h = 4, 6, 8, 10$ for $L = 16, 24, 32, 40$), which accommodates larger cluster sizes and reveals additional lobes with the same near-integer hole mass correspondence. At the same time, the tail of p_m naturally decays for $m \gtrsim 10$ on

finite systems, so very large clusters remain rarer than small ones. By contrast, at $T = 0.025$ the distributions for all L collapse near $m \simeq 12$, consistent with period ≈ 8 stripe order on $W = 4$. Together with the width scan in Fig. 3 of the main text (where width $W = 6$ yields a broader p_m than $W = 4$ at the same area), these observations suggest (as a conjecture for the 2D thermodynamic limit in the low doping regime of the Mott insulator) that increasing system extent supports broader distributions and larger cluster sizes while preserving the characteristic lobe pattern associated with near-integer hole aggregation.

---

[1] F. Verstraete, J. J. Garcia-Ripoll, and J. I. Cirac, Matrix product density operators: Simulation of finite-temperature and dissipative systems, Phys. Rev. Lett. **93**, 207204 (2004).

[2] J. Eisert, M. Cramer, and M. B. Plenio, Colloquium: Area laws for the entanglement entropy, Reviews of modern physics **82**, 277 (2010).

[3] U. Schollwöck, The density-matrix renormalization group in the age of matrix product states, Ann. Phys. **326**, 96 (2011).

[4] I. Peschel, Calculation of reduced density matrices from correlation functions, Journal of Physics A: Mathematical and General **36**, L205 (2003).
